# Electrophysiological Screening for Children With Suspected Auditory Processing Disorder: A Systematic Review

**DOI:** 10.3389/fneur.2021.692840

**Published:** 2021-08-23

**Authors:** Panting Liu, Huiqin Zhu, Mingxia Chen, Qin Hong, Xia Chi

**Affiliations:** ^1^School of Nursing, Nanjing Medical University, Nanjing, China; ^2^Department of Child Health Care, The Affiliated Obstetrics and Gynecology Hospital of Nanjing Medical University, Nanjing Maternity and Child Health Care Hospital, Nanjing, China; ^3^School of Nursing, Nanjing Medical University, Nanjing, China

**Keywords:** auditory processing, auditory processing disorder, electrophysiological testing, children, systematic review, screening

## Abstract

**Objective:** This research aimed to provide evidence for the early identification and intervention of children at risk for auditory processing disorder (APD). Electrophysiological studies on children with suspected APDs were systematically reviewed to understand the different electrophysiological characteristics of children with suspected APDs.

**Methods:** Computerized databases such as PubMed, Cochrane, MEDLINE, Web of Science, and EMBASE were searched for retrieval of articles since the establishment of the database through May 18, 2020. Cohort, case-control, and cross-sectional studies that evaluated the literature for the electrophysiological assessment of children with suspected APD were independently reviewed by two researchers for literature screening, literature quality assessment, and data extraction. The Newcastle–Ottawa Scale and 11 entries recommended by the Agency for Healthcare Research and Quality were used to evaluate the quality of the literature.

**Results:** In accordance with the inclusion criteria, 14 articles were included. These articles involved 7 electrophysiological testing techniques: click-evoked auditory brainstem responses, frequency-following responses, the binaural interaction component of the auditory brainstem responses, the middle-latency response, cortical auditory evoked potential, mismatch negativity, and P300. The literature quality was considered moderate.

**Conclusions:** Auditory electrophysiological testing can be used for the characteristic identification of children with suspected APD; however, the value of various electrophysiological testing methods for screening children with suspected APD requires further study.

## Introduction

Central auditory processing is the perception of auditory information by the central auditory nervous system (CANS) and neurobiological activity in the processing of auditory information and its evoked auditory physiological potentials (1). The American Speech–Language–Hearing Association (ASHA), the American Academy of Audiology (AAA), and the British Society of Audiology (BSA) define perceptual processing deficits of auditory information by the CANS as a central auditory processing disorder (CAPD) (1–3). These children primarily exhibit difficulty with speech-in-noise comprehension, frequently demand repetition, and have associated auditory attention and auditory memory deficits (4). The proposal of an auditory processing deficit has resulted from complaints of hearing problems despite normal audiograms noted in the study by Bocca et al. (5). Auditory problems still occur at 0.5–1% in children with normal peripheral hearing (6). According to Chermak and Musiek, the prevalence of auditory processing deficits in children ranges from 2 to 3% (7). Meanwhile, the presence of auditory processing abnormalities in children with learning difficulties reaches 30–50% (8). Auditory processing deficits are often comorbid with other mental developmental disorders (9–12). Children with speech delay, dyslexia, and ADHD are often accompanied by relatively poor auditory processing skills (13–15), and children with auditory processing deficits may face problems with language, learning, and social communication.

Auditory processing deficits stem from impaired neural function in the CANS (1, 2); meanwhile, the plasticity of the brain structure and function can activate neurons or form effective synaptic connections within the brain (16). Performing top–down auditory training can effectively improve auditory processing in children with auditory processing deficits (17, 18). Therefore, the early identification of children with abnormal auditory processing, followed by an auditory intervention, bears clinical significance.

Current assessment methods used to identify abnormalities in the auditory processing function in children include subjective behavioral tests and objective electrophysiological tests (19). Subjective behavioral tests are divided into verbal and non-verbal tests, such as auditory discrimination tests, temporal processing tests, binaural interaction tests, dichotic speech tests, and monaural low-redundancy speech tests, among others (1). The diagnostic criteria for APD recommended by ASHA are as follows: in the behavioral testing battery, at least two or more behavioral test results, two standard deviations below the mean on either one or both ears (1, 7) or in one test, three standard deviations below the mean on both ears. In addition, several audiologists with extensive experience in the clinical evaluation of auditory processing also reached a consensus on this criterion (8, 20, 21). This criterion assesses auditory processing skills in children by using at least two or more behavioral testing methods while supplying information beyond auditory processing deficits and provides a basis for individualized interventions (2). However, the use of behavioral tests as diagnostic criteria for APD yields inconclusive results (22, 23). Wilson assessed auditory processing in 150 children by using nine sets of diagnostic criteria for behavioral tests; the prevalence of suspected auditory processing disorder (sAPD) ranged from 7.3 to 96.0% (22). No uniform standards have been established in the current screening for auditory processing (1, 2, 24); meanwhile, the sensitivity and specificity of various subjective behavioral tests have not been clarified because of the heterogeneity of auditory processing deficits (7). However, the current gold standard for the diagnosis of CAPD has not been established, leading to controversy over the definition and use of APD (25, 26). The abnormal performance of children with auditory processing deficits in life and the challenges presented are undeniable; thus, effective screening tools for auditory processing have to be further explored.

The AAA practice guidelines indicate the clinical value of auditory electrophysiological testing in screening children for APD (2). Objective electrophysiological testing partly compensates for the inadequacy of behavioral testing, which (i) provides more objective results regardless of the level of language, attention, and cognition of the child and (ii) and owing to the faster development of the CANS (27), electrophysiological testing can be used when unable to cooperate with the completion of behavioral testing and the evaluation of auditory processing in younger children while enabling the early screening of children with sAPD. Owing to its advantages, auditory electrophysiological testing has gained increasing attention from audiologists and clinicians in hearing processing assessment but the clinical application of electrophysiological testing remains more limited because of the lack of supporting evidence. Currently, systematic reviews of behavioral tests for auditory processing screening have been conducted (28, 29); by contrast, no systematic review has been performed on the results of different electrophysiological tests in children with abnormal auditory processing. Thus, the current study provides a supportive basis for selecting screening tools for electrophysiological tests in children with sAPD through a systematic review of the clinical use of different electrophysiological tests in children with abnormal auditory processing.

## Methods

### Search Strategy

Computer searches of the databases PubMed, Cochrane, Medline, Web of Science, and EMBASE were performed from their inception to May 18, 2020 by using the search terms “auditory processing, “auditory processing disorder,” “electrophysiology,” “event-related potential,” “evoked potential,” “auditory evoked response,” “AER,” “auditory brainstem response,” “ABR,” “cortical response” “P300,” “Biomark,” “mismatch negativity response,” “MMN,” “auditory middle latency response,” “AMLR,” “auditory late response,” and “ALR.”

### Inclusion and Exclusion Criteria

The inclusion criteria were as follows: (1) Study subjects included children with APD or children with sAPD who had normal peripheral hearing; (2) The testing method conducted was electrophysiological testing; (3) Study types included cohort, case-control, and cross-sectional studies; and (4) The articles were published in English.

Exclusion criteria: (1) Study subjects included neonates or adults and those diagnosed with the disease or in combination with other mental developmental disorders, such as ADHD, ASD, dyslexia, language impairment; (2) Reviews and republications; and (3) Animal experimental studies.

### Literature Screening, Extraction and Evaluation

In accordance with the inclusion and exclusion criteria, two authors independently screened the literatures. After the duplicate articles were deleted, the literatures were screened by reading the title and abstract of the literatures. For inconclusive literatures, the third author mediated and jointly decided. Finally, the literatures that met the inclusion criteria were retained based on the full reading of the text. Data were extracted independently by two authors and included the following: author, research object, sample size, electrophysiological testing method, results, and conclusion. Two authors used 11 items recommended by the Agency for Healthcare Research and Quality (30) to evaluate the literature quality of the cross-sectional study; a “no” or “unclear” response was assigned a score of “0,” whereas a “yes” response was assigned a score of “1.” The Newcastle-Ottawa Scale (31) was used to evaluate the quality of cohort studies and case-control studies, and a ⋆ was assigned a score of “1” (Table 1).

**Table 1:** Data of selected articles, including study, sample, electrophysiological, results, conclusion and quality score.

**Study**	**Sample**	**Electrophysiological**	**Results**	**Conclusion**	**Quality score**
1. Ankmnal-Veeranna et al. (32)	G1: sAPD (*n* = 108, 5.25–15.7 years, Mean age: 9.63 ± 2.70) G2: TD (*n* = 22, 4.11–16.1 years, mean age:10.71 ± 3.40)	Click ABRs	Children sAPD not significant compared with TD children. However, individual children sAPD showed clinically significant delays	This study provides supportive evidence for the value of click-evoked ABRs in comprehensive auditory processing assessment batteries	AHRQ: 7
2. Abdollahi et al. (33)	Age: 8–12 years *N* = 120 G1: suspected with CAPD (*n* = 60) G2: normal children (*n* = 60) Both groups were sex-matched (40 males and 20 females) and age-matched (9.05 ± 1.25 years)	BIC of MLR	Latency of Pa and Na (ms), Pa–Na amplitude (lv), BIC latency (ms), and BIC amplitude (lv) in children with suspected CAPD were significantly different from those in normal children	MLR and its BIC are clinically available and objective tests that can be used for determining children with suspected CAPD	AHRQ: 6
3. Koravand et al. (34)	Age: 9–12 years *N* = 23 G1: with normal hearing acuity and CAPD (*n* = 10) G2: with normal hearing without CAPD (*n* = 13)	CAEP and MMN	No significant differences in P1 latency and amplitude between groups. Children with CAPD had significant N2 latency prolongation and amplitude reduction. No significant differences in MMN conditions between groups	The N2 response may be a marker of neural deficits in children with CAPD	AHRQ: 6
4. Zakaria and Jalaei (35)	Age:5–9 years (mean = 6.8 ± 3.3 years) *N* = 17 (6 males, 11 females) Healthy and no hearing difficulties	FFR	No significant differences in all FFR results between first and second sessions (*p* > 0.05)	Highly stable FFR results (peak latencies, peak amplitudes, and composite onset measures) over the period of 3 months	NOS: 7
5. Rocha-Muniz et al. (36)	Age:7–15 years (mean = 10 years) N = 27 Abnormal FFR and normal hearing evaluation	FFR	85.15% probability of obtaining deficits on behavioral evaluation of AP in a child with abnormal FFR	FFR in clinical practice as a tool to evaluate AP and assess younger children	NOS: 6
6. Tomlin and Rance (37)	G1: APD (*n* = 27, mean age: 7–12) G2: control group (*n* = 27, mean age:7–12) Had normal hearing	CAEP (P1, N1, P1-N1)	Significant differences in increased P1 and N1 latencies and reduced P1-N1 amplitude in children diagnosed with APD	Immaturity of the CANS as an underlying cause of APD in children	AHRQ: 6
7. Kumar and Singh (38)	Age: 8–12 years G1: Abnormal AP (*n* = 15, 8 males; 7 females, mean age: 9.87 ± 1.35) G2: age-matched typically developing children (*n* = 15, 8 males; 7 females, mean age:9.33 ± 1.45) Had normal hearing sensitivity	FFR (Biomark)	Significant prolongations of wave V and A latencies (*p* = 0.001) and marginal reductions in V/A slope (*p* = 0.052) and amplitude of responses to first formant (*p* = 0.065)	FFR, through the use of BioMARK protocol, could clearly demarcate between children at risk for CAPD and typically developing Children, exhibiting great potential as an electrophysiological tool for the assessment of such individuals	AHRQ: 7
8. Rocha-Muniz et al. (39)	Age: 6–12 years *N* = 75 (had normal hearing) G1: TD (*n* = 25; 8.80 ± 2.08 years; 12 males and 13 females) G2: APD (*n* = 25; 8.72 ± 1.67 years; 18 males and 7 females) G3: SLI (*n* = 25; 7.84 ± 1.77 years; 18 males and 7 females)	FFR	The A wave exhibited superior balance for the APD group (68% specificity, 80% sensitivity, and 74% accuracy)	FFR is a useful test to identify auditory processing disorders	AHRQ: 6
9. Hornickel et al. (40)	Age:3–18 years (mean age: 10.5 years, 14 males 12 females) *N* = 26 (typically-developing children, had normal hearing	FFR	Highly replicable response timing and spectral encoding over the course of 1 year	The FFR may be a unique tool for research and clinical assessment of auditory function, particularly in auditory communication skills	NOS: 6
10. Schochat et al. (41)	Age: 8–14 years *N* = 52 G1: APD children (*n* = 30) G2: normal children (*n* = 22) Had normal hearing	MLR	Before auditory training, the MLR result for the CAPD group exhibited lower C3-A1 and C3-A2 wave amplitudes than those for the control group. After training, the MLR C3-A1 and C3-A2 wave amplitudes of the CAPD group significantly increased	These findings suggest progress in the use of electrophysiological measurements for the diagnosis and treatment of CAPD	AHRQ: 7
11. Roggia and Colares (42)	Age: 9–14 years *N* = 16 G1: APD children (*n* = 8) G2: normal children (*n* = 8) Had normal hearing	MMN	No significant differences in latency and amplitude values among children with APD	The CAPD individuals evaluated showed no changes in MMNf or MMNd MMN could not be considered as a measure of the presence or absence of hearing disorders in CAPD subjects	AHRQ: 6
12. Liasis et al. (43)	G1: sAPD (*n* = 9, 8–12 years, mean 9.5 years, 4 males, 5 females) G2: normal control group (*n* = 9, 8–12 years, mean 10 years, 5 males, 4 females) Had normal hearing	CAEP and MMN	Significantly increased N1 peak latency and a larger peak-to-peak amplitude of the P85−120-N1 and P2-N2 and smaller peak-to-peak amplitude of the N1-P2 in the sAPD children No significant difference in MMN between the control subjects and sAPD	Neurophysiological measures may identify a group of children with specific problems suggestive of APD, in the absence of an obvious structural or functional lesion, who deserve further study to assess whether these findings reflect delayed CNS myelination	AHRQ: 6
13. Delb et al. (44)	Age: 6–12 years *N* = 60 G1: sAPD children (*n* = 17, mean: 8.8 ± 1.6) G2: normal children (*n* = 25, mean: 8.8 ± 1.4) Had normal hearing	BIC of ABR	76% sensitivity and specificity could be achieved	BIC measurements might be of some diagnostic value in CAPD patients	AHRQ: 5
14. Jirsa and Clontz (45)	Age: 9.2–11.6 years *N* = 36 G1: CAPD children (*n* = 18) G2: normal children (*n* = 18)	CAEP and P 300	Significant increases in latency for the N1, P2, and P3 components in the processing disordered group The interpeak latency interval P2-P3 was significantly longer in the clinical group	The long-latency potentials may be useful in the assessment of children with processing disorders	AHRQ: 7

*G, group; TD, typically developing; AP, auditory processing; APD, auditory processing disorder; sAPD, suspected auditory processing disorder; ABR, auditory brainstem responses; BIC, binaural interaction component; MLR, middle-latency response; CAEP, cortical auditory evoked potential; MMN, mismatch negativity; FFR, frequency-following response; AHRQ, Agency for Healthcare Research and Quality; NOS, Newcastle-Ottawa Scale*.

## Result

**Figure 1:**
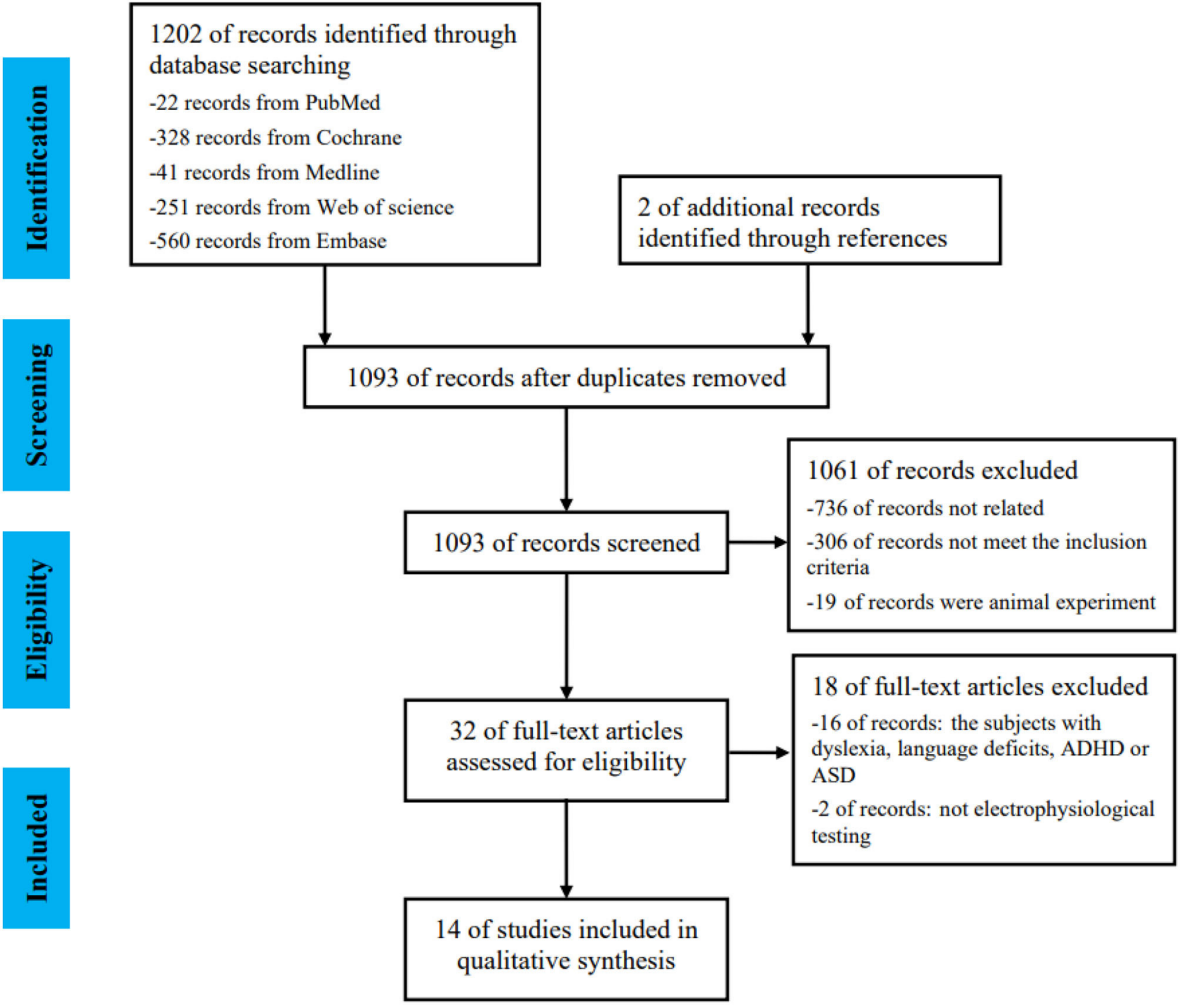
Flowchart of literature screening.

A total of 1,202 articles were retrieved from five databases, as follows: PubMed (22), Cochrane (328), MEDLINE (41), Web of Science (251), and EMBASE (560). In accordance with the inclusion and exclusion criteria, 14 articles were selected using the screening process shown in Figure 1. The 14 articles included 7 electrophysiological tests: click-evoked auditory brainstem responses (click ABR), frequency-following responses (FFR), binaural interaction component of the auditory brainstem responses (BIC of ABR), the middle-latency response (MLR), the cortical auditory evoked potential (CAEP), mismatch negativity (MMN), and P300. All aforementioned electrophysiological tests were conducted on participants aged 5–18 years. The literature quality was moderate.

### Click-Evoked Auditory Brainstem Responses

The click ABR is an objective electrophysiological test widely used to assess hearing threshold and brainstem neural integrity (46, 47). Ankmnal-Veeranna et al. retrospectively compared 108 children with sAPD and 22 normal children for auditory brainstem response recording (32). Recording of click ABR in this study consisted of a slow stimulus (13.3 beats/s) and a fast stimulus (57.7 beats/s) presented to the left and right ears via headphones at an acoustic stimulus of 80 dBnHL, and the typical clinical indexes (latency and interpeak interval of waves I, III, and V) were analyzed. Wave I latencies were significantly longer in children with sAPD than those in normal children (*P* = 0.027), whereas wave III and V latencies were not statistically different between the two groups. No significant difference in the interwave interval was observed between children with sAPD and normal children, except for a prolonged mean interval (>2 standard deviations above normal) in individual children with sAPD. The children with sAPD were less stable than the normal children (*P* = 0.039). The ABR is a useful tool for exploring the integrity of auditory brainstem pathways but has not been widely used for auditory processing assessment because of the lack of supporting evidence (23). In addition, the sAPD children included in the Ankmnal-Veeranna et al. study were not identified by an evaluation with the recommended behavioral testing battery; instead, they were referred solely by physicians, community audiologists, parents, and family friends. They were assessed using the children auditory performance scale (48) and screening identification for targeting educational risk (49). These two subjective scales screen for children with pre-existing auditory problems. Thus, the value of click ABR for auditory processing assessment needs further validation.

### Frequency-Following Response

Frequency-following Response (FFR) is an electrophysiological method that reflects the fidelity and precision of brain encoded sound (50). For many years, FFR has been confused with some terms, such as speech evoked ABR (sABR) and complex sounds evoked ABR (cABR). In order to unify the terms and avoid unnecessary differences among researchers, scholars suggest using a more accurate and appropriate term FFR (50). Therefore, we will use FFR as a substitute for speech evoked ABR (sABR) used by other authors.

FFR has increasingly gained research interest in recent years. Contrary to the traditional click and tone burst, FFR uses the complex syllable /da/ to evoke auditory brainstem response, which is mainly used to analyze the neural activity of the brainstem to speech stimulus (51). The FFR is highly reproducible in young adults (52) and tends to mature in children by age 5 (53). In 2017, Zakaria and Jalaei studied the test-retest reliability of the FFR in normal schoolchildren (35). The test was conducted on 17 healthy children (6 males and 11 females) aged 5–9 years, using 30 dB SL, 40 ms speech sounds /da/ for low-level stimulation. After 3 months, the FFR test was repeated. No significant difference between the first and second FFR test was found (*P* > 0.05). Correlation analysis of the two recordings was performed; the peak latencies, peak amplitudes (V, A, C, D, E, F, O), and composite initiation indexes (V/A duration, V/A amplitude, and V/A slope) of the FFR were highly correlated, particularly the peak latencies of the FFR. In 2012, Hornickel et al. analyzed the speech /da/ evoked FFR in 26 normal children (aged 8–13 years) with an interval of 1 year, collected in a quiet environment and with background noise (sounds spoken by six individuals). Reaction times and spectral coding were found to be highly reproducible over a period of 1 year (40).

The FFR was first used in studies of children with learning disabilities and speech perception deficits (54). In 2014, Rocha-Muniz et al. analyzed the sensitivity, specificity, and efficiency of the FFR for the diagnosis of sAPD (39). The study included 25 children with sAPD and 25 normal children. Children with sAPD had subnormal results on two or more of the four behavioral test battery (the dichotic digits test, staggered spondaic words, speech-in-noise testing and frequency pattern testing) (1). FFR was induced by the syllable /da/, and the waveforms V, A, C, D, E, F, and O were analyzed. The latencies of waves V, A, C, and O were significantly prolonged in children with sAPD, relative to those in normal children. Wave cutoffs were determined by analyzing receiver operating characteristic (ROC) curves. The A-wave sensitivity was 80%, and specificity was 68%. The accuracy of the identification of sAPD was 74%.

In 2016, Rocha-Muniz et al. once again explored the clinical utility of FFR (36), A total of 27 children (aged 7–15 years) with an abnormal FFR were evaluated based on 5 tests of the auditory processing behavior, including sound localization, sequential memory for non-verbal sounds, sequential memory for verbal sounds, speech perception in noise or identification of figures with noise, staggered spondaic word test, frequency and duration pattern tests, and gaps in noise. The results showed that at least one behavioral test abnormality was present in 23 of 27 children with abnormal FFR. This study determined an 85.15% probability of auditory processing deficits in children with abnormal FFR. This calculation is similar to previous findings (39). However, one limitation of this study is the small sample size.

The FFR has been used to explore brainstem encoding of speech signals in children with dyslexia (38, 55), language impairment (56, 57), and learning problems (58–60). However, studies on children with isolated auditory processing abnormalities have rarely been reported. The study of FFR in children with auditory processing abnormalities requires the exclusion of children with higher-order functional deficits, considering that auditory processing abnormalities often coexist with neurodevelopmental disorders, such as language impairment, ADHD, and learning disabilities. In 2015, Kumar et al. first explored BioMark (commercialized FFR) in children with sAPD in whom dyslexia had been excluded (38). The results showed that BioMark waveform morphology was poorer in the presence of children at risk for APD, compared with normal children. Moreover, intergroup comparison indicated that V and A wave latencies were significantly longer in the presence of children at risk for APD (*P* = 0.001). These results suggest the validity of the FFR as an electrophysiological tool for the evaluation of auditory processing. However, Kumar used only the auditory processing screening scale as a tool to identify children with sAPD. Although the scale exhibited high sensitivity and specificity, it also merely stated that these children might be at risk for APD.

### BIC of Auditory Brainstem Responses

The binaural interaction component (BIC) represents the difference between the sum of the monaural evoked potentials and the binaural evoked potentials. Binaural interaction (BI) supports sound localization and auditory behavior under noise and competing acoustic signals at the brainstem level. Electrophysiological testing with BIC is used to evaluate central auditory system abnormalities (61). Gopal and Pierel showed a significant reduction in the BIC amplitude of auditory brainstem responses in children with language disorder and at risk for APD (62). Delb et al. reported on the sensitivity and specificity of the BIC of the auditory brainstem response as a discriminator between children with sAPD and normal children (44). The BIC test was performed on 60 children, divided into the sAPD group (*n* = 17, 8.8 ± 1.6 years) and the normal group (*n* = 25, 8.8 ± 1.4 years) by three behavioral tests: dichotic testing, speech discrimination in noise, and binaural fusion testing. The sensitivity and specificity of the ß component of the BIC as an index for discriminating between children with sAPD and normal children were 76% when subjects underwent 4,000 alternating stimulations of both ears or one ear at 65 dB HL and 60 ms interstimulus intervals.

### Middle-Latency Response

The middle-latency component mainly represents potentials from the thalamus and the primary auditory cortex (63). In 2010, Schochat et al. examined the characteristics of MLRs in 30 children with sAPD, aged 8–14 years (41). At least one ear of the 30 sAPD children's pediatric speech intelligibility test (64), speech-in-noise test (65), staggered spondaic word test (66), the dichotic digit test (67), and the dichotic non-verbal test (65) was lower than the average in two tests. Compared with 22 normal children aged 8–14 years, those with sAPD showed significantly lower Na, PA, and Pb amplitudes at C3-A1 and C3-A2. In the children with sAPD subjected to auditory training for 8 weeks, the amplitudes increased to values similar to those in normal children. Therefore, the MLR is important in identifying children with sAPD and evaluating the efficacy of interventions.

In 2019, Abdollahi et al. identified 60 children with sAPD, aged 8–12 years, in accordance with multiple auditory processing assessment test batteries. These 60 children with sAPD were compared with 60 normal children with respect to the middle-latency response (MLR) and its BIC (33). Significant differences in PA and Na latency (ms), PA and Na amplitude (lv), BIC latency (ms), and BIC amplitude (lv) were determined between children with sAPD and normal children (*P* ≤ 0.001). All waveform latencies were greater and all amplitudes were lower in children with sAPD than in normal children (*P* ≤ 0.001). Therefore, the BIC of MLR and MLR are valuable for identifying potential children with sAPD.

### Cortical Auditory Evoked Potential

As an auditory long-latency response, cortical auditory evoked potential (CAEP) has been used to examine the function of the central auditory system in children with language disorder (68), learning disability (55), and hearing loss (69). The P1-N1 complex components of the CAEP are often used to track the maturation of the central auditory system in hearing-impaired children (70, 71), Tomlin contrasted the CAEP between 27 children with sAPD, aged 7–12 years as determined using AAA-recommended behavioral test battery and 22 matched normal children (37). Presented to the left and right ears as tone burst stimuli at 80 dB HL, 500 Hz, waves P1 and N1 were detected in all children. Compared with normal children, children with sAPD showed increases in P1 and N1 latencies by about 10 ms (*P* < 0.05) and a decrease in P1-N1 amplitude by about 10 μV (*P* = 0.03), whereas no significant difference in the subsequent P2 waves was found between the two groups. P1-N1 complex components may be valid markers of auditory cortex maturity in children with sAPD. In 2003, Liasis et al. compared the ERPs in 9 children with sAPD and 9 normal children in the SCAN/SCAN-A test (43). N1 latency was significantly longer in sAPD children than in normal children, as determined using speech /ba/ as the standard stimulus (*P* = 0.04). On the basis of the wave morphology, the peak-to-peak amplitudes of P85-120-N1 and P2-N2 were larger (*P* = 0.007), whereas the peak-to-peak amplitudes of N1-P2 were smaller in the sAPD children (*P* = 0.004).

The central auditory system processes verbal and non-verbal stimuli differently (72). Consequently, different response patterns can be generated using different types of stimuli. In 2017, Koravand et al. used the “oddball” paradigm for verbal and non-verbal stimuli to record CAEPs (34). The study population included 13 children with sAPD and 10 normal children. The results showed that P1 and N2 waves were observable in all children, and the N2 latency of speech /da/ evoked was longer than that of non-verbal /da/ evoked (*P* < 0.016), and was significantly longer in children with sAPD compared with normal children (*P* < 0.001). During 2 kHz pure tone stimulation, the N2 amplitude was significantly reduced in children with sAPD (*P* < 0.01). However, P1 latency and amplitude were not statistically different between the two groups. The abnormal N2 latency and amplitude may reflect the immaturity of the cerebral cortex in the sAPD children. In addition, the N2 latency evoked by simple stimuli varies from that evoked by complex stimuli. This difference indicates that the central auditory system needs additional time and effort to deal with complex stimuli. A small number of children with hearing processing disorders were included in the study, considering that those with comorbidities, such as children with language and reading difficulties, were excluded.

### Mismatch Negativity

Mismatch negativity (MMN) is an auditory evoked potential with the long-latency response that reflects the early sensory stage of sound processing, the underlying auditory perceptual mechanism (73). It is the difference obtained when auditory event-related potentials obtained with a standard stimulus are subtracted from those obtained with a target stimulus (74). In 2003, Liasis et al. investigated cortical auditory responses in 9 schoolchildren with sAPD (children suspected with APD, based on the clinical presentation and SCAN/SCAN A test) and 9 normal children (43). Speech stimuli consisted of 76% standard stimuli (/ba/) and 24% target stimuli (/da/). No significant differences in MMN latency and peak amplitude were found between children with sAPD and normal children, which was similar to that reported by Roggia and Colares (42). Moreover, no significant differences in the latency or amplitude of MMN were determined between the 8 children with sAPD (as determined by an audiological behavioral assessment in an audiology clinic) and the 8 normal children in whom MMN was evoked with stimuli of different frequencies and durations (standard stimulus, 750 Hz, 100 ms; target stimulus, 1,000 Hz, 50 ms). These results were consistent with the study by Koravand et al. (34), which used non-verbal and verbal stimuli to record CAEPs and involved 23 children, aged 9–12 years. The participants consisted of 10 children with sAPD and 13 normal children. Ten children with sAPD scored below the mean in at least one ear in one test in the behavioral testing battery that included the French adaptation of the Staggered Spondaic Word Test (75), the French adaptation of the Synthetic Sentence Identification-Ipsilateral Competing Message Test (76), the Pitch Pattern Test (77), the Duration Pattern Test (77), and the Random Gap Detection Test (78), with 85% probability for standard stimuli (speech /ba/, non-verbal/ba/, and 1 kHz pure tones) and 15% for target stimuli (speech/da/, non-verbal/da/, and 2 kHz pure tones). The results showed no significant difference in MMN between the two groups. Therefore, the current study cannot yet demonstrate that MMN is a reliable electrophysiological tool for screening children with auditory processing defects.

### P300

P300 is an important endogenous component in the event-related potential (ERP) that responds to high-level cognitive functions in the brain. It is widely studied and has broad clinical applications. Abnormalities in P300 were found in children with cognitive impairment and language disorder, in contrast to normal children (79, 80). In 1990, Jirsa and Clontz applied ERP in the evaluation of children with abnormalities in central auditory processing (45). The study included 8 normal children and 8 sAPD children who scored below the normal response range in at least one ear during behavioral testing (the selective auditory attention competing subtest, pitch pattern sequence test for children-verbal response, pitch pattern sequence test for children-hummed response, and competing sentence test). The 16 aforementioned children showed normal peripheral hearing and normal ABR trajectories. The test stimulus sound at 65 dB HL was presented to both ears in a random sequence, including 20% of the 2 kHz target stimulus and 80% of the 1 kHz standard stimulus. In the test, all children performed the task of distinguishing and counting sounds and were able to produce the P300 waveform. The results indicated that the latencies of N1, P2, and P3 components were significantly increased in the sAPD group. In addition, the peak-to-peak latency P2-P3 in the sAPD children was significantly prolonged, whereas the amplitude of P3 was significantly decreased. These results suggest that ERP, particularly P3, can be used to evaluate children with auditory processing impairment; however, P300 is affected by the attention and cognitive level of children and cannot be elicited in young children.

## Discussion

Auditory processing deficits originate from abnormalities in the development of the central auditory system, with increased emphasis on auditory deficits that are not the result of higher-level cognitive, language, or other related disorders. Auditory processing deficits also fail to cause all learning, language, and social problems (81). The central auditory nervous system is a complex structure with a parallel afferent and a hierarchical afferent (82). The central nervous system has a major function and is responsible for memory, attention, language, and other functions. The auditory system shares neuroanatomical bases and processing with other systems; thus, children with auditory processing deficits often have language (10, 83, 84), attention (85–87), and memory problems (14, 85, 88, 89). The plasticity of the central auditory nervous system renders the auditory training effective in improving auditory processing skills in children with APD (17, 90). Therefore, the early identification and intervention of children with auditory processing defects are important. Auditory processing assessment plays an important role in determining the severity of impaired auditory processing function in children with APD and guiding the construction of individualized intervention protocols (2). Studies have shown that the correlation between the results of the auditory processing behavior test and the electrophysiological test is not considerably high (91). Moreover, the behavioral tests present larger individual differences because auditory behavioral tests require the participation of subjective factors, such as children's language, attention, cognition, and so on. Thus, when children have auditory processing deficits that coexist with other deficits, subjective behavioral tests may be affected by non-auditory factors, influencing the accuracy of the test results. Meanwhile, screening for auditory processing with more than one behavioral test has been associated with enhanced sensitivity but reduced specificity because of the heterogeneity of auditory processing (2). Behavioral tests are more demanding for children, and their test results are more stable only for those aged 7 years and older (2). For younger children, potential auditory processing problems are solved by electrophysiological testing as an objective and reliable means of auditory processing assessment (2), rather than waiting until they reach a testable age. Simultaneously, objective electrophysiological tests are gaining popularity among investigators and clinicians, and ASHA recommends the addition of electrophysiological tests to the evaluation of auditory processing (1). Parthasarathy clarified that auditory electrophysiological measures be included in each central auditory testing battery because they provide objective evidence of central auditory system dysfunction (92). However, the current clinical use of electrophysiological testing remains relatively limited. In the current study, the electrophysiological characteristics of children with sAPD were analyzed using a systematic review of the relevant literature to provide supportive evidence for the selection of electrophysiological assessment tools and evaluation of the efficacy of interventions in children with auditory processing deficits.

The 14 studies that were ultimately considered in this review included 7 electrophysiological tools for auditory processing assessment, which involved the short-latency response, MLR, and the long-latency response of auditory evoked potentials. These responses reflect the auditory functions of the brainstem, thalamus, and auditory cortex of the central auditory system, respectively.

### Short-Latency Response

Auditory brainstem response (ABR) is the most commonly used testing method in short-latency auditory evoked potentials. This systematic review includes seven studies on short-latency auditory evoked potentials, including one study on ABR evoked by simple stimuli, one study on Binaural Interaction Component of ABR, and five studies on FFR evoked by complex speech sounds.

Click ABR is an important index clinically used to detect auditory function and provides information about the functional integrity of brainstem auditory pathways (93, 94). Abnormalities in the latencies and amplitudes of the ABR imply the impaired integrity of the auditory pathways (95, 96). ABR matures earlier (32) and is not affected by the state of consciousness of the child; as such, ABR is clinically more useful for monitoring the integrity of the auditory nerve in infants and young children. Although click ABR may be a useful electrophysiological tool in detecting auditory nerve integrity, its use for the identification of children with APD has not been clearly demonstrated. In the study by Ankmnal-Veeranna et al., click ABR latencies were not significantly prolonged in children with sAPD relative to those in normal children (32). The limitation of this study is that these children with sAPD had no standardized assessment of auditory processing only because of auditory problem referral and through the auditory processing questionnaire evaluation of children with sAPD. The more prominent performance characteristics of these sAPD children are probably not caused by the central auditory nervous system injury but overlap other defects. Therefore, more studies are needed to prove whether click ABR can be used to identify auditory processing defects.

FFR has drawn increasing interest among researchers (97, 98). The advantage of FFR is that compared with ABR evoked by simple stimuli, the syllable /da/ is more compliant with the acoustic characteristics of speech sounds and contains more verbal information (99). The FFR includes the transient components (V, A, C, and O) and periodic components (D, E, and F) (97, 100, 101) and primarily originates from neurons in brainstem nuclei (101). FFR exhibits good test–retest reliability and stability. Zakaria and Hornickel explored the test–retest reliability of the FFR at intervals of 3 months and 1 year, respectively. All showed that the FFR improved stability and can provide distinct information for auditory processing studies and clinical assessment in children (35, 40). Rocha-Muniz conducted studies on the clinical use of the FFR in 2014 and 2016 and confirmed the validity of the FFR as an assessment tool for children with auditory processing deficits (36, 39). Future studies with larger samples are justified to verify the reliability of the results. FFR is widely used in dyslexic children (57, 102, 103). Kumar first explored the value of the FFR as a screening tool for auditory processing when comorbidities were excluded (38). In addition, FFR can be used as a good index to evaluate the effectiveness of auditory training (104).

BICs, which reflect binaural interactions (105–107), have effectively responded to binaural processing functions (108, 109). Binaural processing is one of the important auditory processing behaviors and is associated with sound localization and lateralization and speech recognition in noise. Binaural processing impairment is one of the main manifestations of auditory processing defects. Therefore, the evaluation of binaural interaction is important for screening children at high risk for APD (110, 111), particularly when difficulty in sound localization is observed or when hearing in the presence of noise presents a challenge. The current study of BIC for ABR is extensive and confirms its reliability for assessing binaural processing (112, 113).

### Middle-Latency Response

Only 2 studies on middle-latency auditory evoked potentials in children with sAPD were included in this systematic review, indicating that tests of middle-latency auditory evoked potentials are less frequently used in children with APD.

In the consensus meeting on the diagnosis of APDs in school-age children, the inclusion of ABR and MLR in a minimal APD test battery was proposed (12). MLR is a sensitive indicator of CANS diseases and an important auditory evoked response for identifying auditory processing defects (12, 41, 114). MLR provides information about the integrity of the central auditory system via the primary cortex. MLR is abnormal in children with learning disabilities and language impairment (114, 115). Schochat found that MLR amplitudes were smaller in children with sAPD than in normal children, whereas MLR amplitudes after the intervention increased to levels not significantly different from those of normal children (41). Abdollahi indicated that the BIC of MLR and MLR can be used to identify children with sAPD (33). Compared with that of ABR, the sensitive index for the MLR assessment of central auditory system development is amplitude rather than latency (116); meanwhile, the BIC of MLR is larger and easier to detect than the BIC of ABR. Similar to the ABR, the MLR is not affected by child attention (63). A middle-latency evoked potential has clinical application value in identifying children with sAPD and evaluating intervention effects. However, MLR and BIC of MLR in children with abnormal auditory processing are rarely reported. More studies on the application of middle-latency auditory evoked potentials in children with APD need to be conducted in the future.

### Late-Latency Responses

ERP belongs to late-latency auditory evoked potential, including CAEP, MMN, and P300. ERP refers to the change in brain potential associated with a certain cognitive activity, which reflects the perception and processing ability of the CANS to auditory information (117). This systematic review includes 5 studies on the application of long-latency electrophysiological tests in children with sAPD, with each study potentially involving more than 1 electrophysiological test −4 studies on CAEP, 3 studies on MMN, and 1 study on P300.

The wave latency and amplitude of the CAEP represent the speed and amplitude of central auditory processing (118). Studies showing prolonged latencies and reduced amplitudes indicate a reduced number of contributing neurons, decreased synchronous res*p*onses, altered synaptic density, intracortical myelination, or altered structure/orientation of the auditory pathway (119). The CAEP early components P1 and N1 can be used as markers of auditory cortex maturity in children with auditory processing deficits (37). Unlike CAEP late components, P1 and N1 are passive and do not require the involvement of child attention (120). Liasis noted that the latency and morphology of the N1 wave differ in children with auditory processing deficits and normal children (43). The N1 wave is associated with the sudden appearance or occasional alteration in the frequency of stimulus sounds (121) and may reflect the variable sensitivity of the auditory cortex and cortical activity inside (122). The first component of the N1 wave is the onset of analysis of sensory information and may reflect the initiation of attention and memory formation (122). The P2 wave and the late component of the CAEP are not generated by the auditory cortex but by several sensory modalities (123). Other studies have shown that the source of the N2 wave may be related to the inhibitory response (124). Koravand et al. proposed that the N2 wave can be used as an effective discriminator of sAPD (34). Therefore, children with sAPD can potentially have an inhibitory processing defect.

Passively evoked MMN can provide objective measures for auditory discrimination and the automatic processing of perception (125). Contrary to P300, the MMN was not be affected by subject attention. MMN can be used to assess the central auditory system function in children with dyslexia and autism spectrum disorder (126, 127); however, the value of MMN as a screen in children with sAPD has not been demonstrated.

The endogenous component P300, which is produced in areas other than subcortical structures and temporal lobes, is involved in the attention to and recognition of stimulus sound differences. P300 may also be related to neural activities, such as the processing of sequence information, short-term memory, or decision-making (128). P300 can be used as a meaningful indicator to identify children at risk for APD. As reported by Jirsa and Clontz, children with sAPD showed significantly longer P300 latencies and significantly lower amplitudes compared with normal children (45). In addition, the P300 test can sensitively reflect changes in auditory processing ability (129), providing a reliable basis for the evaluation of auditory processing function and intervention efficacy. P300 maturation is influenced by the developmental level of the child, and its latency decreases with age; meanwhile, elicitation of the P300 wave requires active child participation, such as performing tasks to focus attention, and results are influenced by child attention, cognition, and psychological factors. Thus, abnormal P300 can occur in cases of sAPD but may also be observed in children with cognitive, attention, and other higher-order disorders. Therefore, the interpretation of P300 results in children with auditory processing deficits needs multidisciplinary collaboration to identify children with mental development disorders, such as language impairment, ADHD, and ASD. More electrophysiological studies of long-latency responses in children with auditory processing abnormalities are also needed.

A systematic review of the use of electrophysiological testing at different latencies in children with APD or sAPD showed that electrophysiological testing effectively identified children with auditory processing deficits, aiding the determination of the site and the origin of abnormalities of the central auditory nervous system. The short-latency auditory evoked potentials have been more extensively studied in children with APD, particularly the FFR. The central auditory nervous system function at different levels, from the brainstem to the cerebral auditory cortex, is reflected separately by short- to late-latency electrophysiological responses exhibiting possible abnormalities in the electrophysiological test results at different latencies in children with auditory processing deficits. These results may validate the role of both bottom-up and top-down factors in auditory information processing. The transfer of auditory information is not simply a step-by-step, hierarchically relayed structure of the auditory pathway (20). According to the ease of language underlying (ELU) model (130), when auditory signals are poor or listening under noise, increased auditory cognitive effort is necessary, with more than usual attention and memory involved in the comprehension of language. Therefore, for children with auditory processing abnormalities, electrophysiological tests at different latencies may be required clinically to assess the degree of impairment of the auditory processing function.

However, although the ASHA recommended diagnostic criteria for APD [the results were two standard deviations below the mean in at least one ear on two or more auditory behavioral tests (1)] have been recognized by AAA (2) and many experienced audiologists (20, 131, 132), no unified gold standard for the diagnosis of APD has been established. Therefore, the criteria used to identify children with APD or children with sAPD also varied among the 14 included studies. Two studies (33, 38) used only the children auditory performance scale (48) and screening identification for targeting educational risk (49) and the screening checklist for auditory processing (SCAP) (133) to assess auditory processing abnormalities. Only 3 studies (34, 39, 41) followed the diagnostic criteria recommended by ASHA to identify children with APD. Two studies (44, 45) used less than a certain percentage to define children with APD. Two studies (36, 37) used the criterion of a deficit in at least one of the auditory processing behavioral tests to evaluate children with APD. Additional studies (33, 42, 43) have employed other modalities to identify children with APD. Although children with poor performance in auditory behavioral tests in these studies usually have poor electrophysiological test results, the electrophysiological characteristics of APD children may vary based on different diagnostic criteria. The conclusions drawn for children with sAPD do not necessarily apply to children diagnosed with APD (134); therefore, the conclusions obtained in this study may be more biased toward children with sAPD, which is also one of the limitations of this study. More studies need to be conducted for validation (135). Before then, controversial issues regarding the diagnostic criteria of APD is a critical first step to resolve. The BSA practice guidelines state that the establishment of a gold standard for the diagnosis and management of APD is highly recommended (136). Although many studies have confirmed that behavioral tests and electrophysiological tests can effectively identify damage to the central auditory nervous system in patients with APD, new, more precise, and effective tests for auditory processing still need to be developed (2).

Since APD is often comorbid with other disorders of mental development, such as language disorders, learning disabilities, and ADHD, among others. Behavioral tests used to identify APD often require the involvement of the child's language, attention, and cognition. Thus, it is of particular interest to consider when the results of auditory behavioral tests are poor in children with APD comorbid with other developmental disorders. Whether it is the problem of auditory processing itself or the result of other mental developmental disorders remains inconclusive. This systematic review was aimed at assessing the discriminative value of different methods of electroacoustic testing in children with APD or sAPD. With this objective considered, this review excludes studies in which the subjects were comorbid with other mental developmental disorders. Therefore, some equally important reports may not have been included in the study, such as (62, 137–139). However, compared with other diseases, APD clinically coexists with other psychodevelopmental disorders (10, 12) in more children. Thus, more studies on the electrophysiological characteristics of children with APD combined with different psychodevelopmental disorders need to be performed in the future. Moreover, the diagnosis of children with APD is a complex process (135, 140) and requires the multidisciplinary involvement of audiologists during differential diagnosis, in conjunction with psychologists, speech pathologists, developmental behaviorists, and educators.

## Conclusion

A systematic review of the 7 electrophysiological characteristics of children with sAPD suggests that auditory electrophysiological tests are valuable in identifying children with abnormal auditory processing. FFR has been widely studied, and its clinical application value has been confirmed. The clinical application value of middle- and late-latency physiological potentials in screening children with abnormal auditory processing needs more research for verification. Owing to the complexity of the central auditory system and the heterogeneity and comorbidity of auditory processing defects, the identification of auditory processing defects requires behavioral tests combined with electrophysiological tests of different latency responses. It also requires multidisciplinary collaboration in the differential diagnosis and intervention of auditory processing defects. In addition, the auditory electrophysiological characteristics of children with different mental developmental disorders need to be further examined. Standardized electrophysiological testing data of children in different regions and ages have to be established to provide a basis for the evaluation of the test results.

## Limitations

No consensus has been reached regarding the diagnostic criteria for APD. Different auditory behavioral tests and measures were used to identify children with sAPD in the 14 included studies, although children with poorer performance in the behavioral tests generally showed poorer electrophysiological test results. However, the electrophysiological characteristics of children with sAPD identified in accordance with different diagnostic criteria may be more variable. Therefore, the electrophysiological test results of children with sAPD may not be applicable to all children with APD. Moreover, the study excluded the study of children with APD who are comorbid with other mental development disorders, possibly some equally important reports were excluded, and analyses of the discriminative value of different electrophysiological tests for auditory processing characteristics in children with different mental developmental disorders are needed in the future. Because the patterns presented by the results of the included studies were different, and not all studies could get a quantitative result, meta-analysis of the included studies could not be performed, so more high-quality clinical trial studies are needed to provide strong evidence. For electrophysiological testing, the stability of the testing tool, testing environment, and child status may affect the test results, and electrophysiological testing has the disadvantage of higher examination cost, which may limit the clinical applications of electrophysiological testing. Therefore, new auditory processing evaluation tools with enhanced sensitivity and specificity, which are suitable for clinical application and promotion, still need to be developed.

## Data Availability Statement

The original contributions presented in the study are included in the article/supplementary material, further inquiries can be directed to the corresponding author/s.

## Author Contributions

PL: conceptualization, methodology, and writing—original draft. HZ: data curation. MC: formal analysis. QH: writing—review and editing, supervision, project administration, and funding acquisition. XC: supervision, project administration, and funding acquisition. All authors contributed to the article and approved the submitted version.

## Conflict of Interest

The authors declare that the research was conducted in the absence of any commercial or financial relationships that could be construed as a potential conflict of interest.

## Publisher's Note

All claims expressed in this article are solely those of the authors and do not necessarily represent those of their affiliated organizations, or those of the publisher, the editors and the reviewers. Any product that may be evaluated in this article, or claim that may be made by its manufacturer, is not guaranteed or endorsed by the publisher.
